# Phosphorylation of cysteine string protein-α up-regulates the frequency of cholinergic waves *via* starburst amacrine cells

**DOI:** 10.1017/S0952523822000013

**Published:** 2022-05-11

**Authors:** Ching-Feng Chen, Rita R. Wo, Chien-Ting Huang, Tzu-Lin Cheng, Juu-Chin Lu, Chih-Tien Wang

**Affiliations:** 1 Institute of Molecular and Cellular Biology, National Taiwan University, Taipei, Taiwan; 2 Department of Physiology and Pharmacology, College of Medicine, Chang Gung University, Taoyuan, Taiwan; 3 Division of Endocrinology and Metabolism, Department of Internal Medicine, Chang Gung Memorial Hospital, Linkou, Taoyuan, Taiwan; 4 Department of Life Science, National Taiwan University, Taipei, Taiwan; 5 Neurobiology and Cognitive Science Center, National Taiwan University, Taipei, Taiwan; 6 Genome and Systems Biology Degree Program, National Taiwan University and Academia Sinica, Taipei, Taiwan

**Keywords:** cysteine string protein, PKA-mediated phosphorylation, cholinergic waves, starburst amacrine cells, retinal ganglion cells

## Abstract

During the first postnatal week in rodents, cholinergic retinal waves initiate in starburst amacrine cells (SACs), propagating to retinal ganglion cells (RGCs) and visual centers, essential for visual circuit refinement. By modulating exocytosis in SACs, dynamic changes in the protein kinase A (PKA) activity can regulate the spatiotemporal patterns of cholinergic waves. Previously, cysteine string protein-α (CSPα) is found to interact with the core exocytotic machinery by PKA-mediated phosphorylation at serine 10 (S10). However, whether PKA-mediated CSPα phosphorylation may regulate cholinergic waves *via* SACs remains unknown. Here, we examined how CSPα phosphorylation in SACs regulates cholinergic waves. First, we identified that CSPα1 is the major isoform in developing rat SACs and the inner plexiform layer during the first postnatal week. Using SAC-specific expression, we found that the CSPα1-PKA-phosphodeficient mutant (CSP-S10A) decreased wave frequency, but did not alter the wave spatial correlation compared to control, wild-type CSPα1 (CSP-WT), or two PKA-phosphomimetic mutants (CSP-S10D and CSP-S10E). These suggest that CSPα-S10 phosphodeficiency in SACs dampens the frequency of cholinergic waves. Moreover, the level of phospho-PKA substrates was significantly reduced in SACs overexpressing CSP-S10A compared to control or CSP-WT, suggesting that the dampened wave frequency is correlated with the decreased PKA activity. Further, compared to control or CSP-WT, CSP-S10A in SACs reduced the periodicity of wave-associated postsynaptic currents (PSCs) in neighboring RGCs, suggesting that these RGCs received the weakened synaptic inputs from SACs overexpressing CSP-S10A. Finally, CSP-S10A in SACs decreased the PSC amplitude and the slope to peak PSC compared to control or CSP-WT, suggesting that CSPα-S10 phosphodeficiency may dampen the speed of the SAC-RGC transmission. Thus, *via* PKA-mediated phosphorylation, CSPα in SACs may facilitate the SAC-RGC transmission, contributing to the robust frequency of cholinergic waves.

## Introduction

During a critical period of visual circuit refinement, vertebrate retinas display correlated, patterned, spontaneous activity with unique spatiotemporal properties, so-called retinal waves. In the first week of postnatal rodents, retinal waves are initiated by neurotransmitter release from cholinergic neurons starburst amacrine cells (SACs), thus termed cholinergic waves (Feller et al., 1996; Zheng et al., 2006; Ford et al., 2012). These waves propagate across the entire ganglion cell layer (GCL), consisting of mainly presynaptic SACs and postsynaptic retinal ganglion cells (RGCs, the retinal output neurons). To date, cholinergic waves are found essential for activity-dependent refinement of visual circuits (Blankenship & Feller, 2010; Kirkby et al., 2013). Advanced *in vivo* evidence has confirmed that the patterned spontaneous activity in visual centers initiates from developing retinas, justifying the importance of cholinergic waves to the refinement of global visual circuits (Ackman et al., 2012).

Cholinergic waves are initiated from spontaneous, periodic depolarizations in developing SACs. By activating voltage-gated Ca^2+^ channels, Ca^2+^ influx into SACs can bind the Ca^2+^ sensor protein such as Synaptotagmin I (Syt I) to trigger Ca^2+^-dependent exocytosis, allowing neurotransmitters to be released and further received by neighboring SACs and RGCs. The core exocytotic machinery is the *s*oluble *N*-ethylmaleimide-sensitive factor *a*ttachment protein *re*ceptor (SNARE) complex, composed by *sy*napto*b*revin (Syb) (also termed *v*esicle-*a*ssociated *m*embrane *p*rotein/VAMP), *s*yn*t*a*x*in (Stx), and *s*y*n*aptosome-*a*ssociated *p*rotein of 25 kDa (SNAP-25/SN25) (Sudhof & Rizo, 2011). Remarkably, previous studies showed that periodic oscillations in PKA activity profoundly regulate the spatiotemporal properties of cholinergic waves (Dunn et al., 2006), suggesting the key PKA substrate may involve in regulating wave patterns. Among three SNARE proteins and Syt I, only SN25 can serve as a PKA substrate (Risinger & Bennett, 1999). Consistently, our previous study showed that the spatiotemporal properties of cholinergic waves are regulated by PKA-mediated SN25 phosphorylation in developing SACs (Hsiao et al., 2019). Furthermore, these changes in wave patterns are sufficient to regulate visual circuit refinement, such as eye-specific segregation of retinogeniculate projection (Hsiao et al., 2019). Hence, *via* switching the phosphorylation state by PKA, SN25 in SACs can regulate the patterns of cholinergic waves and sculpt developing visual circuits.

Dual effects have been observed in the PKA regulation of cholinergic waves. On one hand, transiently high PKA activity displays in the middle quiescence of inter-wave intervals (Dunn et al., 2006), suggesting that phosphorylation of certain PKA substrate(s) may restrict the wave occurrence. This effect can be addressed by down-regulation of cholinergic waves *via* PKA-mediated SN25 phosphorylation in SACs. On the other hand, bath-application of the PKA inhibitor (Rp-cAMPS or H-89) reduces the frequency of cholinergic waves (Stellwagen et al., 1999; Huang et al., 2014), suggesting that phosphorylation of some other PKA substrate(s) may increase the wave occurrence. However, to date it is unclear which PKA substrate(s) may up-regulate cholinergic waves *via* SACs. Our previous study has shown that a presynaptic protein, cysteine string protein-α (CSPα), increases the rate of exocytosis by PKA-mediated phosphorylation at serine 10 (S10) in secretory cells (Chiang et al., 2014). Coincidentally, CSPα can interact with SN25 *in vitro* and *in vivo* (Sharma et al., 2011, 2012). These lines of evidence led to the hypothesis that CSPα likely serves as a PKA substrate to up-regulate the wave activity, through facilitating SAC transmission in the developing retina. However, direct evidence is absent to support this hypothesis.

In this study, by combining immunostaining, qPCR, cell type-specific molecular perturbation, live imaging, and whole-cell patch-clamp recordings, we show that in developing SACs, CSPα phosphodeficiency at S10 may dampen the synaptic strength and wave properties, suggesting that presynaptic CSPα may up-regulate cholinergic waves *via* PKA-mediated phosphorylation.

## Materials and methods

### Plasmid information

DNA fragments encoding rat wild-type CSPα1 (pCMV-*Csp*-IRES2-*egfp*), its phosphodeficient mutant (pCMV-*Csp-*S10A-IRES2-*egfp*), or its phosphomimetic mutants (pCMV-*Csp-*S10D-IRES2-*egfp*; pCMV-*Csp-*S10E-IRES2-*egfp*) were obtained from our previous study (Chiang et al., 2014). To target the expression specifically to SACs, these DNA fragments were subcloned into pmGluR2-IRES2-*egfp* (designated Ctrl, hereinafter) (Chiang et al., 2012; Huang et al., 2014; Hsiao et al., 2019) using *Bgl*II and *Not*I, yielding pmGluR2-*Csp*-IRES2-*egfp* (designated CSP-WT, hereinafter), pmGluR2-*Csp-*S10A-IRES2-*egfp* (designated CSP-S10A, hereinafter), pmGluR2-*Csp-*S10D-IRES2-*egfp* (designated CSP-S10D, hereinafter), and pmGluR2-*Csp-*S10E-IRES2-*egfp* (designated CSP-S10E, hereinafter) (Figs. 2, 3A, 3B, and 4). To verify the ectopic gene expression after *ex vivo* electroporation (Fig. 3D and 3E), CSP and its phosphodeficient mutant were constructed into the pmGluR2-HA-IRES2-*egfp* vector (designated HA-Ctrl) (Hsiao et al., 2019), yielding pmGluR2-HA-*Csp*-IRES2-*egfp* (designated HA-CSP-WT) and pmGluR2-HA-*Csp*-S10A-IRES2-*egfp* (designated HA-CSP-S10A) in Fig. 3D and 3E.

**Fig. 1.**
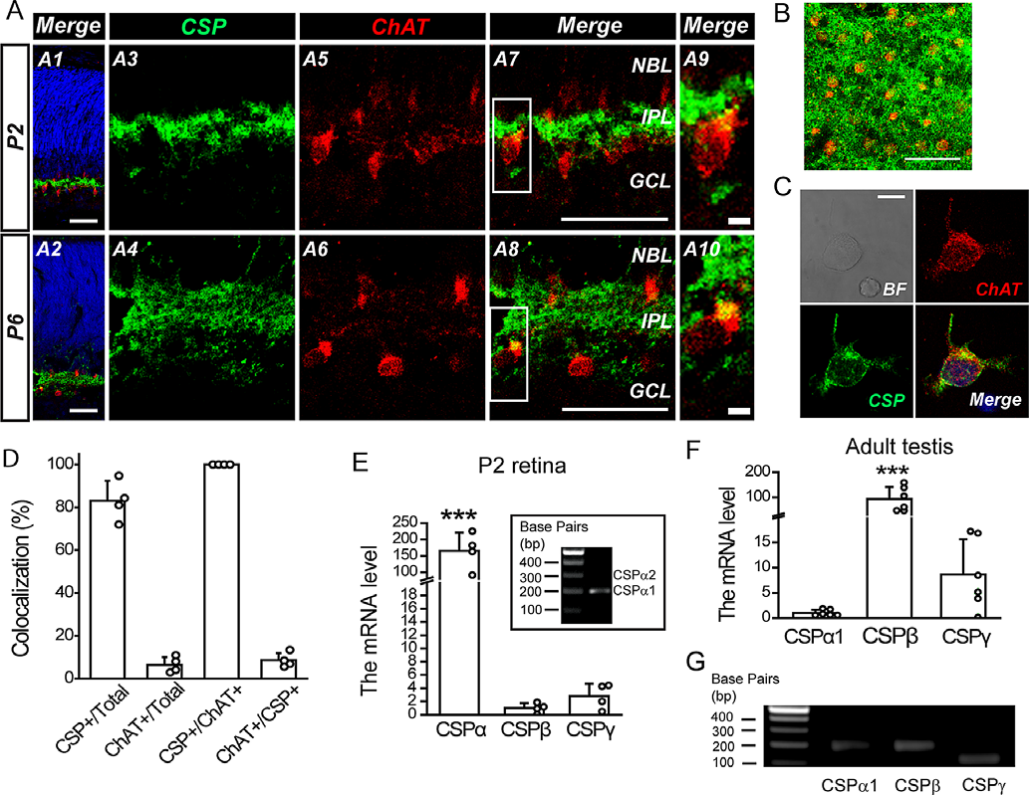
CSPα1 is expressed in developing rat SACs during the first postnatal week. (**A**) Immunostaining of CSP (green), choline acetyltransferase (ChAT, the SAC marker; red), or DAPI (blue) in retinal cross-sections from P2 and P6 rats. The colocalization signals were shown in yellow. GCL, ganglion cell layer; IPL, inner plexiform layer; NBL, neuroblast layer. **A9** and **A10**, Magnification of white boxes in **A7** and **A8**. Scale bars, 50 (**A1** *–* **A8**) and 5 *μ*m (**A9** and **A10**). (**B**) Immunostaining of CSP (green) and ChAT (red) in the IPL from the P2 whole-amount retina (1.5-*μ*m *z*-section). Scale bar, 50 *μ*m. (**C**) Representative immunostaining of CSP (green), ChAT (red), or DAPI (blue) in a dissociated P4 rat SAC (1.5-*μ*m *z*-section). BF, bright field. Scale bar, 7.5 *μ*m. (**D**) The ratios were calculated for the cells displaying the CSP and/or ChAT immunoreactivity in an imaged region consisting of ~20 dissociated P4 retinal cells. Data were from four confocal images. CSP+/Total, the ratio for all dissociated cells with CSP immunoreactivity; ChAT+/Total, the ratio for all dissociated cells with ChAT immunoreactivity; CSP+/ChAT+, the ratio for all SACs with CSP immunoreactivity; ChAT+/CSP+, the ratio for all CSP-expressing cells as SACs. The cutoff intensity was 4.43 for CSP immunoreactivity and 1.9 for ChAT immunoreactivity. The average intensity was 37.15 ± 19.36 (mean ± s.d., *n* = 4) for CSP immunoreactivity and 7.84 ± 2.5 (mean ± s.d., *n* = 4) for ChAT immunoreactivity. (**E**) The endogenous mRNA levels of CSP isoforms (CSPα, CSPβ, and CSPγ) in intact P2 rat retinas. ***P < 0.0001 for CSPα *versus* CSPβ and CSPα *versus* CSPγ, One-way ANOVA with Student–Newman–Keuls *post hoc* test; *n* = 4 retinas from four pups. *Inset panel*, DNA gel electrophoresis after RT-qPCR experiments. (**F**) The endogenous mRNA levels of CSP isoforms (CSPα1, CSPβ, and CSPγ) in intact adult rat testes. ***P < 0.0001 for CSPα1 *versus* CSPβ and CSPβ *versus* CSPγ, One-way ANOVA with Student–Newman–Keuls *post hoc* test; *n* = 6 testes from three rats. For **D**–**F**, circles beside columns represent data from individual samples. For **E** and **F**, the mRNA levels normalized to the mean level of CSPβ and CSPα1, respectively. (**G**) DNA gel electrophoresis after RT-qPCR experiments validated the replicons based on these isoform-specific primers as positive control.

**Fig. 2.**
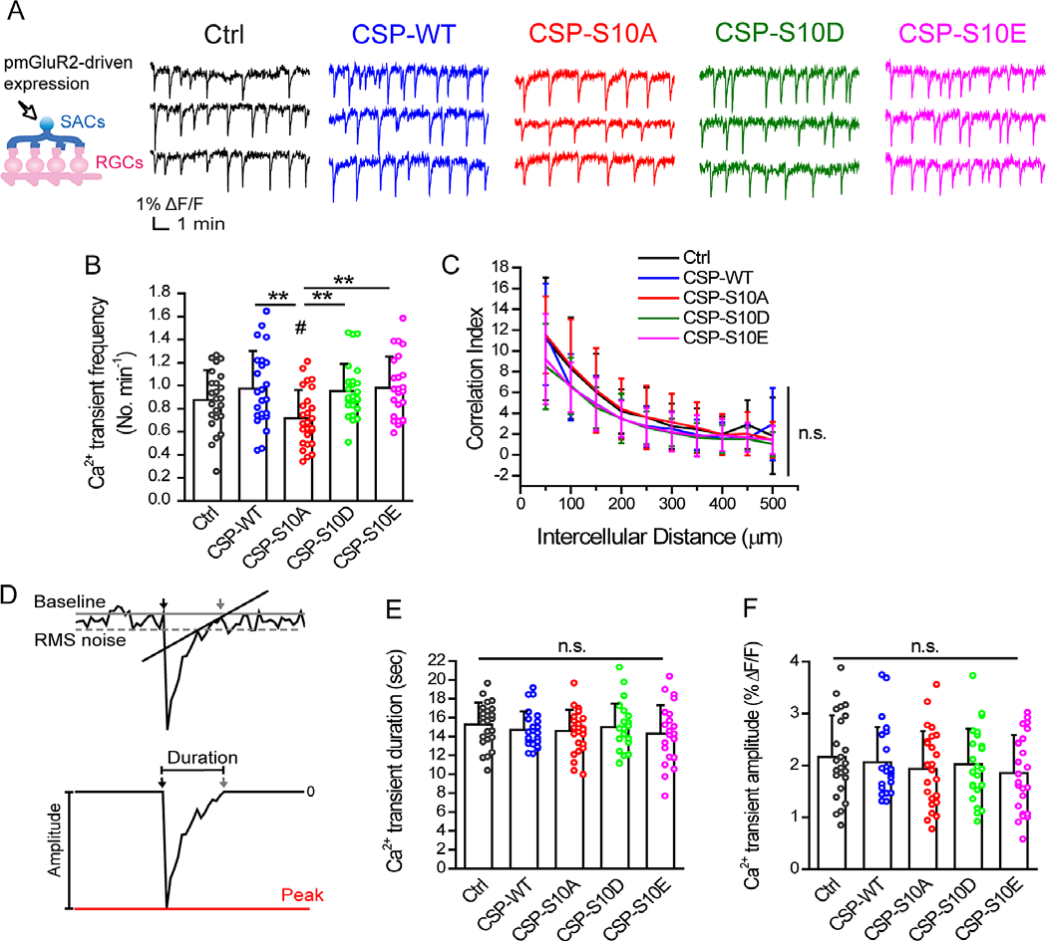
SAC-specific expression of CSP-S10A reduces the frequency of spontaneous Ca^2+^ transients. (**A**) Molecular perturbation in P1-P2 rat SACs was performed by the mGluR2 promoter-driven expression, including Ctrl (pmGluR2-IRES2-*egfp*), CSP-WT, CSP-S10A, CSP-S10D, and CSP-S10E. Representative spontaneous Ca^2+^ transients after SAC-specific expression were shown in the 10-min live imaging. (**B**) Ca^2+^ transient frequency. **P = 0.0015, One-way ANOVA with Student–Newman–Keuls *post hoc* test. #*P* = 0.0305 for CSP-S10A *versus* Ctrl; no significance for any other groups *versus* Ctrl (*P* = 0.26 for CSP-WT *vs.* Ctrl; *P* = 0.28 for CSP-S10D *vs.* Ctrl; *P* = 0.18 for CSP-S10E *vs.* Ctrl), two-tailed Student’s unpaired *t*-test. (**C**) The intercellular correlation of spontaneous Ca^2+^ transients. n.s., no significance (*P* = 0.07), Repeated measures ANOVA with Tukey *post hoc* test. (**D**) A single Ca^2+^ transient. *Top*, the starting point (black arrows) and the end point (gray arrows) were defined by the criteria in section “Event definition.” The baseline (the gray solid line); The RMS noise (the gray dash line). *Bottom*, the Ca^2+^ transient duration and amplitude (ranging from the baseline to the peak, the red line). (**E**) Ca^2+^ transient duration. n.s., no significance, One-way ANOVA with Student–Newman–Keuls *post hoc* test (*P* = 0.81) or two-tailed Student’s unpaired *t*-test (*P* = 0.36 for CSP-WT *vs.* Ctrl; *P* = 0.29 for CSP-S10A *vs.* Ctrl; *P* = 0.66 for CSP-S10D *vs.* Ctrl; *P* = 0.21 for CSP-S10E *vs.* Ctrl). (**F**) Ca^2+^ transient amplitude. n.s., no significance, Kruskal–Wallis test with Dunn *post hoc* test (*P* = 0.82), Mann–Whitney test (*P* = 0.53 for CSP-WT *vs.* Ctrl), or two-tailed Student’s unpaired *t*-test (*P* = 0.30 for CSP-S10A *vs.* Ctrl; *P* = 0.53 for CSP-S10D *vs.* Ctrl; *P* = 0.17 for CSP-S10E *vs.* Ctrl). For **B**, **C**, **E**, and **F**, Data were acquired from 440 cells, 44 imaged regions, 24 retinal explants, and 10 pups for Ctrl; 480 cells, 48 imaged regions, 24 retinal explants, and 8 pups for CSP-WT; 467 cells, 47 imaged regions, 26 retinal explants, and 9 pups for CSP-S10A; 470 cells, 47 imaged regions, 25 retinal explants, and 8 pups for CSP-S10D; 480 cells, 48 imaged regions, 24 retinal explants, and 9 pups for CSP-S10E. Circles beside columns represent data from individual retinal explants.

**Fig. 3.**
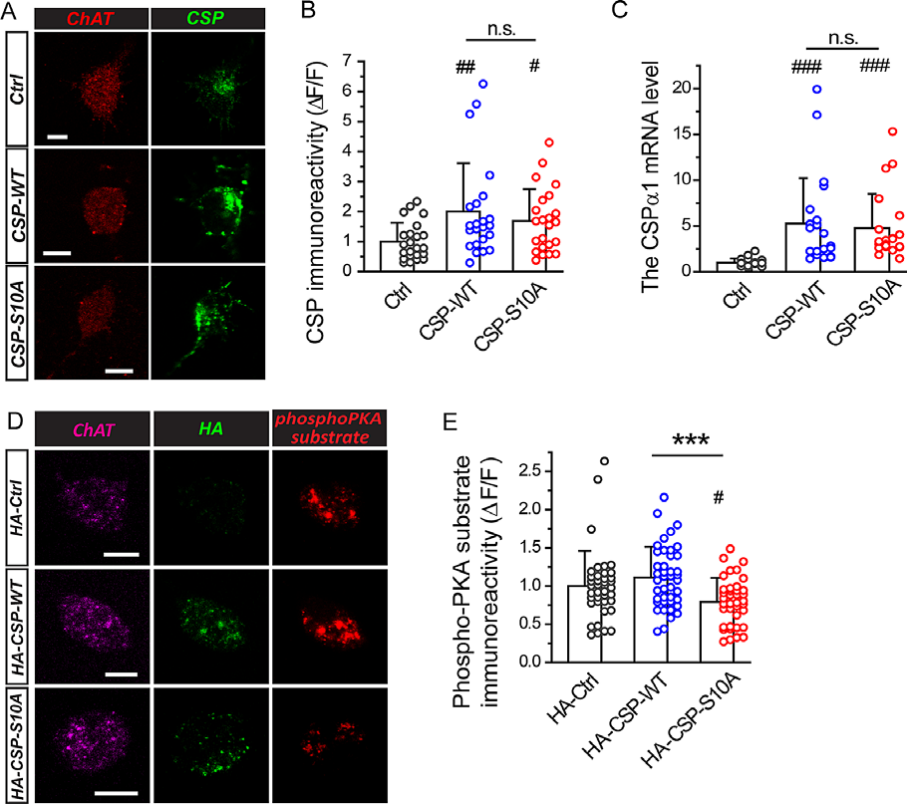
PKA-mediated phosphorylation is reduced by expressing CSP-S10A in developing SACs. (**A**) Immunostaining of ChAT (red) or CSP (green) in dissociated SACs expressing Ctrl (pmGluR2-IRES2-*egfp*), CSP-WT, or CSP-S10A (1.5-*μ*m *z*-section). Scale bars, 5 *μ*m. (**B**) Quantification of the CSP immunoreactivity in transfected SACs. ^##^*P* = 0.0057 *versus* Ctrl, Mann–Whitney test. ^#^*P* = 0.011 *versus* Ctrl, two-tailed Student’s unpaired *t*-test. n.s., no significance (*P* = 0.83), Mann–Whitney test. *n* = 23 SACs for each transfection group. (**C**) The relative mRNA levels of CSPα1 in transfected retinas expressing Ctrl, CSP-WT, or CSP-S10A. ^###^*P* < 0.0001 *versus* Ctrl; n.s., no significance (*P* = 0.73), Mann–Whitney test. *n* = 21, 22, and 21 transfected retinas for Ctrl, CSP-WT, and CSP-S10A, respectively. (**D**) Immunostaining of ChAT (magenta), HA (green), or phospho-PKA substrate (red) in dissociated SACs expressing HA-Ctrl (pmGluR2-HA-IRES2-*egfp*), HA-CSP-WT, or HA-CSP-S10A (1.5-*μ*m *z*-section). Scale bars, 5 *μ*m. (**E**) Quantification of the phospho-PKA substrate immunoreactivity in transfected SACs. ***P = 0.0002, two-tailed Student’s unpaired *t*-test. ^#^*P* = 0.03 for HA-CSP-S10A *versus* HA-Ctrl; no significance (*P* = 0.14) for HA-CSP-WT *versus* HA-Ctrl, Mann–Whitney test. *n* = 39, 42, and 38 SACs for HA-Ctrl, HA-CSP-WT, and HA-CSP-S10A, respectively. For **B**, **C**, and **E**, circles beside columns indicate the data from individual SACs.

**Fig. 4.**
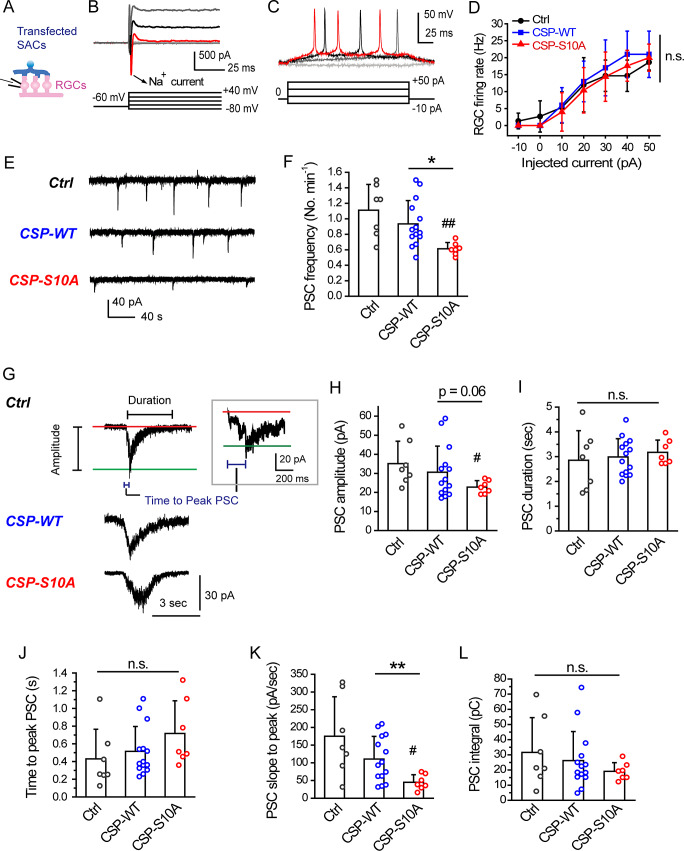
CSP-S10A dampens the strength of transmission from developing SACs. (**A**) Whole-cell patch-clamp recordings were performed in the RGCs proximal to transfected SACs expressing Ctrl (pmGluR2-IRES2-*egfp*), CSP-WT, or CSP-S10A. (**B**) The RGC was identified by quickly activated Na^2+^ currents (arrow) upon depolarizing pulses. (**C**) Action potentials were induced in a RGC by injecting various sizes of current pulses (−10 to +50 pA for 200 ms; Δ*I* = 20 pA). (**D**) The firing rate of action potentials in a RGC after SAC-specific expression. n.s., no significance (*P* = 0.15), Repeated measures ANOVA with Tukey *post hoc* test. Data were obtained from three to five RGCs, two retinal explants, and two pups. (**E**) The wave-associated postsynaptic currents (PSCs) were recorded in RGCs proximal to transfected SACs for 6 min. (**F**) PSC frequency. *P = 0.013, two-tailed Student’s unpaired *t*-test. ^##^*P* = 0.0024 for CSP-S10A *versus* Ctrl; no significance (*P* = 0.24) for CSP-WT *versus* Ctrl, two-tailed Student’s unpaired *t*-test. (**G**) Amplitude, duration, and time to the peak (blue) in representative PSCs (by the criteria in section “Event definition”). Inset, the enlarged PSC event on a different scale from the left panel. The baseline (red); The peak line (green). (**H**) PSC amplitude. ^#^*P* = 0.036 for CSP-S10A *versus* Ctrl; no significance (*P* = 0.44) for CSP-WT *versus* Ctrl; no significance (*P* = 0.06) for CSP-WT *versus* CSP-S10A, two-tailed Student’s unpaired *t*-test. (**I**) PSC duration. n.s., no significance (*P* = 0.79 for CSP-WT *vs.* Ctrl; *P* = 0.53 for CSP-S10A *vs.* Ctrl; *P* = 0.50 for CSP-WT *vs.* CSP-S10A), two-tailed Student’s unpaired *t*-test. (**J**) Time to peak PSC. n.s., no significance (*P* = 0.32 for CSP-WT *vs.* Ctrl; *P* = 0.07 for CSP-S10A *vs.* Ctrl; *P* = 0.17 for CSP-WT *vs.* CSP-S10A), Mann–Whitney test. (**K**) Slope to peak PSC. **P = 0.0029, two-tailed Student’s unpaired *t-*test. ^#^*P* = 0.023 for CSP-S10A *versus* Ctrl; no significance (*P* = 0.19) for CSP-WT *versus* Ctrl, two-tailed Student’s unpaired *t*-test. (**L**) PSC integral. n.s., no significance (*P* = 0.69 for CSP-WT *vs.* Ctrl; *P* = 0.26 for CSP-S10A *vs.* Ctrl; *P* = 0.44 for CSP-WT *vs.* CSP-S10A), Mann–Whitney test. For (**F**) and (**H–L**), Data were acquired from seven RGCs, four retinal explants, and four pups for Ctrl; 14 RGCs, five retinal explants, and five pups for CSP-WT; seven RGCs, four retinal explants, and four pups for CSP-S10A. Circles beside columns represent data from individual retinas.

### Animals

Postnatal (P1–P6) Sprague–Dawley (SD) rat pups with either sex were used in this study. All procedures were performed in accordance with protocols approved by the institutional animal care and use committees of National Taiwan University (NTU). The pups were bred from their own mothers (from BioLASCO, Taipei, Taiwan; *ad libitum* access to food and water) in the individually ventilated cages under well-controlled conditions (12:12 light/dark cycle with light on 7 AM; 22 ± 1°C). All rat pups were deeply anesthetized before decapitation with isoflurane to minimize suffering with all efforts.

### Retinal explant culture and transfection

Postnatal retinas were obtained from SD rat pups (P1–P2) and transfected by *ex vivo* electroporation (Chiang et al., 2012; Huang et al., 2014; Hsiao et al., 2019). Briefly, after decapitation, the retinas were isolated and cut into three pieces in dissection buffer [1 × HBSS (Gibco), 10 mM HEPES, and 0.35 g/L NaHCO_3_, pH 7.35]. The retinal pieces (retinal explants) were attached onto nitrocellulose membranes (Millipore) with the GCL up. Retinal explants were incubated with serum-free culture medium-adult (SFCM-A) [Neurobasal-A (Gibco), 0.6% glucose, 2 mM l-glutamine (Sigma), 1 × B27 (Gibco), 10 mM HEPES, 1 mM sodium pyruvate (Gibco), 2.5 *μ*g/ml insulin (Sigma), 100 *μ*g/ml penicillin (Gibco), 100 units/ml streptomycin (Gibco), and 6 *μ*M forskolin (Sigma)] at 35°C in a humidified atmosphere of 5% CO_2_ and supplied with fresh culture medium daily. To perform transfection, retinal explants were incubated in dissection buffer containing plasmid DNA (200 ng DNA/*μ*L) at RT for 10 min. The retinal explants were placed in the gap of horizontal platinum electrodes (4 mm) (Chiang et al., 2012) filled with 400 *μ*L DNA-containing buffer and transfected by electroporation (27 V, 50 ms of pulse duration, 2 square pulses at 1-s interval; BTX ECM830, Harvard Apparatus). After electroporation, retinal explants were cultured in SFCM-A with forskolin and fed with fresh culture medium daily until 72 h post transfection for further experiments (Chiang et al., 2012; Huang et al., 2014; Hsiao et al., 2019).

### Immunostaining

For immunostaining of retinal cross-sections, anesthetized postnatal pups (P2 or P6) were perfused with 1 × phosphate-buffered saline (PBS; 136.89 mM NaCl, 2.68 mM KCl, 10.14 mM Na_2_HPO_4_, and 1.76 mM KH_2_PO_4_, pH 7.4) and 4% paraformaldehyde (PFA). Isolated eyeballs were kept in 4% PFA at 4°C 30 min and in 30% sucrose at 4°C for overnight, followed by preservation in optimal cutting temperature (OCT) gel. Retinal cross-sections (16 *μ*m) were prepared with a cryostat (Leica CM1850), placed on poly-lysine-coated slides, and blocked at RT for 1 h in donkey serum blocking solution [DBS; 3% donkey serum (Jackson Lab) and 0.5% Triton X-100 in 1 × PBS]. Retinal sections were incubated at 4°C overnight with the primary antibodies in 1% DBS [goat anti-choline acetyltransferase (ChAT; Millipore AB144P, 1:200) and rabbit anti-CSP (Millipore AB1576, 1:1000)], washed with PBS for 1 h, incubated at RT for 2 h with the secondary antibodies in 1% DBS [donkey-anti-goat IgG conjugated to Alexa Fluor 568 (Invitrogen, 1:400) and donkey-anti-rabbit IgG conjugated to Alexa Fluor 488 (Invitrogen, 1:400)], and further stained with 4′,6-diamidino-2-phenylindole (DAPI; Sigma) at RT for 15 min. The cross-sections on slides were sealed by coverslips with Fluoromount.

For immunostaining of whole-mount retinas, P2 whole-mount retinas were fixed in 4% PFA at RT for 30 min and then washed with 1 × PBS for 1 h. After fixation, the explants were blocked and permeabilized in DBS at RT for 1 h, and then incubated with the primary antibodies in 1% DBS (the same as described above) at 4°C for 2 days and washed with PBS for 1 h. The secondary antibodies in 1% DBS (the same as described above) were added to the explants at 4°C for 2 h. The explants were washed with PBS for 1 h. Finally, the explants were mounted on glass slides with Fluoromount and sealed with coverslips.

For immunostaining of dissociated retinal neurons, isolated retinal neurons were acquired from P2 or P4 pups (Grozdanov et al., 2010), cultured on the coated coverslips, fixed with 4% PFA at RT for 20 min, and washed with 1 × PBS for 20 min. After fixation, the cells were permeabilized with 0.1% Triton X-100 for 10 min and blocked in 3% DBS with 0.1% Triton X-100 at RT for 1 h. After blocking, the neurons were incubated at 4°C overnight with the primary antibodies [goat anti-ChAT and rabbit anti-CSP; goat anti-ChAT, rabbit anti-phospho-PKA substrate (Cell Signaling 9624, 1:200), and mouse anti-HA (Covance MMS-101P, 1:800)], washed with PBS for 1 h, incubated at RT for 2 h with the secondary antibodies in 1% DBS [donkey-anti-goat IgG conjugated to Alexa Fluor 647 (Invitrogen, 1:400), donkey-anti-rabbit IgG conjugated to Dylight 549 (Jackson Immuno Research, 1:400), and donkey-anti-mouse IgG conjugated to Alexa Fluor 488 (Invitrogen, 1:400)], and further stained with DAPI at RT for 15 min. Finally, the neurons on coverslips were mounted on slides with Fluoromount.

Images were acquired by laser-scanning confocal microscopy (Leica TCS SP5 spectral) in *z*-series, consisting of one plane (1.5-*μ*m thickness) for whole-mount retinas/dissociated cells and 8–11 planes for retinal cross-sections (Chiang et al., 2012; Huang et al., 2014; Hsiao et al., 2019). For quantification of immunoreactivity, images were imported into MetaMorph software to quantify the changes in fluorescence intensity for each cell, with subtraction and normalization by the same-sized background (Δ*F*/*F*) (Arndt-Jovin et al., 1985; Huang et al., 2014; Hsiao et al., 2019; Webster et al., 2020). The levels of fluorescence intensity were normalized to the mean level of the Ctrl group (Fig. 3B and 3E). Data were further analyzed by Excel and Origin 8.

### Reverse-transcriptase quantitative PCR

The RNA sample from whole-mount retinas or testes was extracted by TRIzol reagent (Invitrogen). Briefly, the samples were homogenized by adding 1 ml TRIzol reagent and phase-separated by 0.2 ml chloroform. After centrifugation at 12,000 *g* at 4°C for 15 min, the RNA was extracted in the upper aqueous phase (~0.6 ml) and then precipitated with 0.5 ml isopropanol. The RNA-containing solution was centrifuged at 12,000 *g* at 4°C for 10 min, and the supernatant was discarded. The RNA pellets were washed once with 75% ethanol, followed by centrifugation at 12,000 *g* at 4°C for 10 min. After complete removal of the supernatant, the RNA pellets were air-dried at RT for 20 min upon transparency, dissolved in 20 *μ*L of diethyl pyrocarbonate (DEPC)-treated water, and incubated at 60°C for 10 min. The RNA samples were stored at −80°C for further experiments. The cDNAs were synthesized from RNAs using the ProtoScript II First Strand cDNA Synthesis kit (New England BioLabs). Reverse-transcriptase quantitative PCR (RT-qPCR) was performed on the cDNA samples using the LabStar SYBR qPCR kit (TAIGEN Bioscience Corporation). The primers specific for the transcripts of CSPα1, CSPα2, CSPβ, CSPγ, or β-actin were obtained from our previous study (Chiang et al., 2014). To detect the ectopic HA-CSP (WT or S10A) expression, the forward primer was designed to target the HA-tag (5′-CCA TGT ACC CAT ACG ATG TTC CAG-3′, underlined) and the reverse primer was designed to target CSPα1 (5′-TCT GCA GCC TCT GGG TTA TC-3′), yielding the replicon length as 200 base pairs. The SYBR fluorescence data were collected during the extension step of each cycle (totally 40 cycles of 95°C for 3 s, 60°C for 45 s, and 72°C for 30 s) and transformed into the cycle threshold (Ct) values using the qPCR machine (Qiagen Rotor-Gene Q) and supplemental software (Qiagen Rotor-Gene Series Software 1.7). The ΔCt was obtained by subtracting the Ct of the reference gene (β-actin) from the Ct of the target gene. The ΔΔCt was obtained by subtracting the average of the ΔCt of CSPβ (Fig. 1E), CSPα1 (Fig. 1F), or Ctrl (Fig. 3C) from the ΔCt of the other target gene or transfection group. The relative expression levels of the target genes were calculated as 2^(−ΔΔCt)^.

### Live Ca^2+^ imaging

Transfected retinal explants were transferred into SFCM-A without forskolin overnight before imaging, and then incubated for 30–60 min in the Ca^2+^ indicator-containing medium (10 *μ*M fura-2-AM, 0.02% pluronic acid, and 1% DMSO in the SFCM-A without forskolin). During image acquisition, retinal explants were perfused with artificial cerebrospinal fluid (ACSF) (in mM: 119 NaCl, 26.2 NaHCO_3_, 2.5 KCl, 1.0 K_2_HPO_4_, 1.3 MgCl_2_, 2.5 CaCl_2_, and 11 D-glucose) bubbled with 95% O_2_/5% CO_2_ warmed to 30°C. Imaging experiments were performed under a 20 × water immersion objective (Olympus BX51WI) with excitation at 380 nm and emission at 510 nm. Fluorescence images were captured using a CCD camera (CoolSnap HQ2, PhotoMetrics) at 1-s intervals for 10 min (Chiang et al., 2012; Huang et al., 2014; Hsiao et al., 2019). Wave-associated Ca^2+^ transients were acquired from the fluorescence changes across 601 time frames, each with background subtraction using the MetaMorph software (Molecular Devices). To analyze the characteristics of spontaneous Ca^2+^ transients, the photobleached baseline was corrected using our Igor (WaveMetrics) procedure as previously reported (Chiang et al., 2012; Huang et al., 2014; Hsiao et al., 2019). The fluorescence value (*F*) in each Ca^2+^ transient was subtracted from the baseline (*F*_0_) and further divided by the baseline [(*F − F*_0_)/*F*_0_] to produce the trace of Δ*F*/*F.* Further, the written Igor procedure would automatically pick the peaks of Ca^2+^ transients (see the detail in section “Event definition”). All data of Ca^2+^ transients were averaged from each cell, averaged across 10 randomly selected cells out of one imaged region, and then averaged from two imaged regions out of one retinal explant. Finally, the mean data for each group were averaged from all retinal explants transfected with the same gene. Further analysis was performed by Excel and Origin 8 (OriginLab) (Chiang et al., 2012; Huang et al., 2014; Hsiao et al., 2019).

Spatial correlation of spontaneous Ca^2+^ transients was evaluated by the correlation index (CI) (Wong et al., 1993; Torborg et al., 2005) according to the following equation:




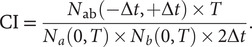


*N*_ab_ is the transient number for which cell *b* exhibits in a time window ± Δ*t* (3 s) from cell *a.* *N_a_* and *N_b_* are the total numbers of transients exhibited by cells *a* and *b*, respectively, during the total imaging time (*T*, 600 s). The averaged CI values were computed from the same distance group and plotted against the intercellular distance as previously described (Chiang et al., 2012; Huang et al., 2014).

### Electrophysiology

Whole-cell patch-clamp recordings were performed on visualized RGCs (60 × water-immersion objective, Olympus) in transfected retinal explants with SAC-specific expression. During recordings, retinas were continuously perfused with oxygenated ACSF at 30°C as described previously (Huang et al., 2014; Hsiao et al., 2019). Borosilicate glass pipettes (WPI#PG52151-4) were pulled (Narishige PC-10) to a tip resistance of ~5.5 MΩ when filled with a pipette solution [98.3 K-gluconate, 1.7 KCl, 0.6 EGTA, 5 MgCl_2_, 40 HEPES, 2 Na_2_-ATP, 0.3 Na-GTP (in mM), pH 7.25 with KOH]. Recordings were made using an Axopatch 200B patch-clamp amplifier with Digidata 1440A interphase (Molecular Devices). Data were acquired and analyzed with the pClamp10 software (Molecular Devices). In whole-cell voltage-clamp recordings, the spontaneous wave-associated postsynaptic currents (PSCs) (filtered at 1 kHz and digitized at 5 kHz) were recorded at a holding potential of −72 mV (the liquid junction potential as −12 mV) (Fig. 4E–4L). The induced current responses were also measured using the protocols indicated elsewhere (Fig. 4B). Whole-cell current-clamp recordings were used to measure the action potential firings (filtered at 5 kHz and digitized at 10 kHz) (Fig. 4C and 4D). In successful recordings, gigaohm seals were obtained within 30 s, and the ratios of access resistance to input resistance were 5–15%. Data from wave-associated PSCs were averaged over all events from one cell. The final data for each group were averaged across all cells transfected with the same gene (Huang et al., 2014; Hsiao et al., 2019).

### Event definition

For individual Ca^2+^ transients, the definitions of the duration and amplitude were presented in Fig. 2D. The written Igor procedure would automatically pick the peaks of Ca^2+^ transients, with their fluorescence intensity twofold greater than the root-mean-square (RMS) noise (about 0.25% Δ*F*/*F*) as reported previously (Chiang et al., 2012). The RMS noise was measured from the fluorescence trace starting with 30 s before the peak to 50 s following the peak. The starting point (*x*_0_, *y*_0_) of an event (Fig. 2D, black arrows) was defined by the time point where the first derivative was zero right before the peak (i.e., *dy/dx* = 0, where *y* was the fluorescence changes in %Δ*F*/*F* and *x* was the recording time in seconds). To define the end point (*x*′, *y*′) of an event (Fig. 2D, gray arrows), a line was first drawn to connect the time points where the fluorescence trace was back to the range within the RMS noise. The end point was defined by the time point with the minimal fluctuation of fluorescence (i.e., *y*′ − *y*_0_ = minimum). Finally, the Ca^2+^ transient duration was defined as the interval between the starting and end points. The Ca^2+^ transient amplitude was defined as the fluorescence change from the baseline to peak (shown as the red line in Fig. 2D).

For individual wave-associated PSCs, the definitions of amplitude, duration, and time to the peak were presented in Fig. 4G. The RMS noise was measured from the trace between 10 s prior to the event and 15 s following the event. The potential PSC events were picked with their current response 2.5-fold greater than the RMS noise. Further, to distinguish from the miniature synaptic events (minis), wave-associated PSCs were identified by the slow inward current (~s) with the large event integral (>3.5 pC). The amplitude of wave-associated PSCs was defined as the current change from the baseline (Fig. 4G, the red line) to the peak, that is, the maximal inward current that lies in the middle of the recording noise by taking the average across 10 data points (Fig. 4G, the green line). The PSC duration was defined as the interval between the trace left from (the starting point) and returned to (the ending point) within the RMS noise of the baseline. The time to the peak PSC was defined as the interval between the starting point and the peak of an event. The slope to peak PSC was individually calculated from the PSC amplitude divided by the time to peak, reflecting the transmission rate reaching to the maximal postsynaptic response. The PSC integral was calculated from the area of individual events, reflecting the amount of input signal received by postsynaptic cells.

### Statistics

Data were presented as means with standard deviation (s.d.) (OriginLab). Statistical significance ≥3 groups (CSP-WT and three CSP phosphomutants) was evaluated by one-way ANOVA with Student–Newman–Keuls *post hoc* test for the parametric method or by Kruskal–Wallis test with Dunn *post hoc* test for the nonparametric method (*P < 0.05; **P < 0.01; ***P < 0.001). Statistical significance between control and the other transfection group was evaluated by two-tailed Student’s unpaired *t*-test as a parametric method or by Mann–Whitney method as a nonparametric method (^#^*P* < 0.05; ^##^*P* < 0.01; ^###^*P* < 0.001 *vs.* Ctrl/HA-Ctrl). Repeated measures ANOVA with Tukey’s multiple comparison *post hoc* test was used to evaluate significant differences in the correlation index and RGC firing rate (InStat 3, GraphPad). n.s., no significance (*P* > 0.05).

## Results

### CSPα1 is expressed in SACs during the period of cholinergic waves

To investigate the role of PKA-mediated CSPα phosphorylation in regulating cholinergic waves, we first detected whether developing SACs expressed CSP during the cholinergic wave period. By immunostaining P2 or P6 retinal cross-sections, we found that CSP immunoreactivity mainly localized to the inner plexiform layer (IPL) (i.e., developing cholinergic synapses), as demonstrated by the immunoreactivity of the SAC marker (choline acetyltransferase; ChAT) (Fig. 1A). Moreover, we immunostained the P2 whole-mount retina and imaged at a 1.5-*μ*m *z*-section (Fig. 1B). We found that the CSP immunoreactivity was mainly found around the SAC somata (labeled by the ChAT immunoreactivity) in the narrow *z*-section of IPL, suggesting that CSP may localize to the synapses around SACs. Consistently, we found the CSP immunoreactivity in dissociated developing SACs (Fig. 1C). Further quantification (Fig. 1D) showed that the CSP immunoreactivity could be detected in 83% of dissociated cells in the imaged region (CSP+/Total). Even though only 7% of total dissociated cells were apparently SACs that exhibited the detectable ChAT immunoreactivity (ChAT+/Total), all these SACs displayed the detectable CSP immunoreactivity (100% in CSP+/ChAT+). Instead, only 8.5% of CSP-immunoreactive cells were apparently SACs (ChAT+/CSP+), suggesting that non-SAC cells, such as RGCs, might also express CSP that contributed to the CSP immunoreactivity observed in the IPL. Although the CSP immunoreactivity is also found in other types of retinal cells, CSP expression in developing SACs may play a role in regulating cholinergic transmission *via* SACs.

CSP consists of three isoforms (*α*, *β*, and *γ*) and CSPα consists of two splicing variants (*α*1 and *α*2) (Brown et al., 1998; Fernandez-Chacon et al., 2004). To determine the CSP isoform(s) that were expressed in developing rat retinas, we performed RT-qPCR. As the results, CSPα1 was the isoform solely expressed in developing rat retinas during the stage of cholinergic waves (Fig. 1E). By contrast, adult rat testes mainly expressed two other isoforms (CSPβ and CSPγ) (Fig. 1F) as previously reported (Brown et al., 1998; Fernandez-Chacon et al., 2004), validating the effectiveness of these isoform-specific primers (Fig. 1G).

### CSP-S10 phosphodeficiency in SACs dampens the frequency of spontaneous Ca^2+^ transients associated with cholinergic waves

To further determine how CSPα regulated cholinergic waves by PKA-mediated phosphorylation, we manipulated the CSPα expression level or the CSPα phosphorylation states in SACs using the SAC-specific promoter (the type II metabotropic glutamate receptor promoter, pmGluR2), as validated from previous studies (Watanabe et al., 1998; Chiang et al., 2012; Huang et al., 2014; Hsiao et al., 2019). Postnatal retinas were transfected with the control vector (designated Ctrl, hereinafter), wild-type CSPα1 (designated CSP-WT, hereinafter), CSPα1 harboring a PKA-phosphodeficient mutation (replacing serine 10 with alanine; designated CSP-S10A, hereinafter), or CSPα1 harboring a PKA-phosphomimetic mutation (replacing serine 10 with aspartate or glutamate; designated CSP-S10D or CSP-S10E, hereinafter) (Chiang et al., 2014). To detect alterations in the properties of cholinergic waves, we performed live Ca^2+^ imaging to measure wave-associated spontaneous Ca^2+^ transients following SAC-specific expression of CSPα or its phosphomutants (Fig. 2A). Spontaneous, correlated Ca^2+^ transients in individual cells revealed cholinergic waves in the RGC layer of transfected retinal explants (Fig. 2A). Ca^2+^ transient frequency remained similar by expressing Ctrl, CSP-WT, CSP-S10D, or CSP-S10E in developing SACs (Fig. 2B), suggesting that the endogenous level of CSPα expression or of PKA-mediated CSPα phosphorylation is sufficient in maintaining the robust frequency of cholinergic waves. By contrast, Ca^2+^ transient frequency was significantly reduced by expressing CSP-10A in SACs compared to all other groups (Fig. 2A and 2B). These results suggest that PKA-mediated CSPα phosphorylation at S10 contributes to robust wave frequency.

Since PKA-mediated CSPα phosphorylation in SACs regulates wave frequency, we next determined whether CSPα or it phosphomutants in SACs could alter the spatial properties of cholinergic waves. To address this, we constructed the pair-wise correlation in neighboring cell pairs across the same imaged regions (Fig. 2C) (Chiang et al., 2012; Huang et al., 2014). We found that expressing CSPα or it phosphomutants in SACs did not change the correlation index across various intercellular distances (Fig. 2C), suggesting that PKA-mediated CSPα phosphorylation in SACs may not alter the spatial correlation of cholinergic waves.

Ca^2+^ transient size represents the level of Ca^2+^ influx into neurons during individual waves. To further determine whether Ca^2+^ transient size was regulated by PKA-mediated CSPα phosphorylation in SACs, we quantified the duration and amplitude of spontaneous Ca^2+^ transients (Fig. 2D–2F). As the results, the duration and amplitude of single Ca^2+^ transients were comparable among all groups, suggesting that PKA-mediated CSPα phosphorylation in SACs did not affect the size of spontaneous Ca^2+^ transients.

### PKA-mediated phosphorylation is reduced by expressing CSP-S10A in developing SACs

Previous studies showed that SAC-specific perturbation became effective after 2 days post transfection (Chiang et al., 2012; Huang et al., 2014). To detect the level of CSPα expression, we performed immunostaining on dissociated SACs at 72 h post transfection (Fig. 3A). The level of CSP immunoreactivity was significantly higher in SACs expressing CSP-WT or CSP-S10A compared to Ctrl (pmGluR2-IRES2-*egfp*) (Fig. 3A and 3B). Consistently, qPCR analysis suggested that the retinas transfected with CSP-WT or CSP-S10A displayed significantly higher expression of CSPα1 mRNA compared to Ctrl (Fig. 3C). Moreover, compared with Ctrl, the SACs transfected with CSP-WT or CSP-S10A displayed about twofold CSP intensity (Fig. 3A and 3B), suggesting that these transfected CSP constructs contributed to roughly equal protein amount to the endogenous CSP.

Next, we measured the phosphorylation level of PKA substrates in dissociated SACs (Fig. 3D). Following the SAC-specific expression of HA-CSP constructs, we first determined ectopic expression of HA-CSP constructs by qPCR analysis. The ratios of the relative mRNA levels for the ectopic CSP (HA-CSPα1) *versus* all detectable CSP (CSPα1) were similar in P2 retinas expressing HA-CSP-WT or HA-CSP-S10A (HA-CSP-WT: 0.48 ± 0.22, *n* = 3; HA-CSP-S10A: 0.65 ± 0.34, *n* = 4. *P* = 0.46, two-tailed Student’s unpaired *t*-test.). Further, we performed immunostaining in transfected SACs and justified that HA-CSP-WT and HA-CSP-S10A were efficiently expressed in the SACs (Fig. 3D). However, we found that the level in the phospho-PKA substrate immunoreactivity was significantly lower in SACs expressing HA-CSP-S10A compared to HA-Ctrl or HA-CSP-WT (Fig. 3D and 3E), suggesting that the phosphorylated level of PKA substrates was significantly reduced in SACs expressing CSP-S10A. Thus, these results suggest that the SACs expressing CSP-S10A may display the relatively low level of PKA phosphorylation. Given that SACs expressing CSP-S10A also displayed the reduced wave frequency (Fig. 2B), these results are consistent with the previous report (Dunn et al., 2006) showing that the dampened wave frequency is correlated with the decreased PKA activity.

### CSPα-S10 phosphodeficiency in SACs dampens synaptic transmission to RGCs

Since CSPα-S10 phosphodeficiency in SACs reduced wave frequency, we next determined whether the effects attributed to a decrease in synaptic transmission from SACs to RGCs. To address this, we performed whole-cell patch-clamp recordings on postsynaptic RGCs after SAC-specific expression (Fig. 4A). First, to identify RGCs nearby transfected SACs, we performed whole-cell voltage-clamp recordings. Upon supra-threshold depolarizations, the recordings from RGCs revealed quickly inactivated, large Na^+^ currents, justifying the cells we recorded were RGCs (Fig. 4B).

Next, to examine whether presynaptic CSPα may alter the intrinsic excitability of postsynaptic RGCs, we performed whole-cell current-clamp recordings from RGCs and measured the firing rate of action potentials by stepwise current pulses (Fig. 4C). We found that SAC-specific expression of Ctrl, CSP-WT, or CSP-S10A did not affect the RGC firing rate, suggesting that presynaptic overexpression of CSPα or its phosphodeficient mutant cannot alter the intrinsic excitability of postsynaptic RGCs (Fig. 4D).

Further, to determine if presynaptic expression of CSP-S10A decreased synaptic transmission from SACs to RGCs, we detected the periodicity of wave-associated inputs received by postsynaptic RGCs. RGCs displayed spontaneous, wave-associated, compound PSCs following SAC-specific CSP expression (Fig. 4E). Comparing with Ctrl or CSP-WT, overexpressing CSP-S10A in SACs significantly decreased the frequency of wave-associated PSCs (Fig. 4F). These results suggest that CSPα-S10 phosphodeficiency in SACs may reduce the periodicity of wave-associated inputs received by postsynaptic RGCs. Together, PKA-mediated CSPα phosphorylation in SACs may up-regulate the periodicity of inputs that RGCs receive during waves, without altering the RGC’s intrinsic membrane properties.

Finally, to examine whether CSPα-S10 phosphodeficiency in SACs could reduce the level of input signals that RGCs receive during waves, we detected the characteristics of individual wave-associated PSCs (Fig. 4G), including peak amplitude (Fig. 4H), duration (Fig. 4I), the time reaching to peak (Fig. 4J), the slope reaching to peak (Fig. 4K), and integral (Fig. 4L). In comparison with Ctrl or CSP-WT, expressing CSP-S10A in SACs did not change duration, the time reaching to peak, and integral (Fig. 4I, 4J, and 4L), but reduced peak amplitude (Fig. 4H) and the slope reaching to peak (Fig. 4K). Note that the slope reaching to peak is acquired from the peak amplitude divided by the time reaching to peak, reflecting the rate reaching to the maximal postsynaptic response for individual events. Hence, a reduction in the slope reaching to peak represents a decrease in the speed of SAC-RGC transmission. Together, these results suggest that CSPα-S10 phosphodeficiency in SACs may just dampen the speed, rather the amount, of input signals that RGCs receive during waves. Thus, through PKA-mediated S10 phosphorylation, CSPα in SACs may facilitate synaptic transmission from SACs to RGCs.

## Discussion

In this study, we found that developing SACs and IPL mainly express CSPα1. The endogenous level of PKA-mediated CSPα phosphorylation contributes to the robust frequency of cholinergic waves. Overexpressing the PKA-phosphodeficient CSP mutant (CSP-S10A) in SACs decreases the frequency of wave-associated spontaneous Ca^2+^ transients. Moreover, the SACs expressing CSP-S10A exhibit a decreased level of the phospho-PKA substrates. Furthermore, from electrophysiological recordings, overexpressing CSP-S10A in SACs reduces the periodicity of the SAC-RGC transmission and dampens the speed of input signals that RGCs receive during waves. These results suggest that PKA-mediated CSPα phosphorylation at S10 in SACs is important for maintaining cholinergic waves. Therefore, during the critical developmental period, CSPα can serve as a PKA substrate that up-regulates cholinergic waves.

The cAMP-PKA signaling displays dynamic spatial–temporal distributions in the developing neurons (Dunn & Feller, 2008). Multiple cAMP-dependent mechanisms have been found to diversely regulate the spatiotemporal properties of retinal waves. For example, the long-lasting after-hyperpolarizations in SACs are mediated by the cAMP-sensitive, Ca^2+^-activated K^+^ channels, which can regulate the wave refractory period and set the upper limit of wave frequency (Zheng et al., 2006). In addition to the regulation by cAMP, PKA can also affect cholinergic waves by phosphorylation of the downstream substrates. Developing retinal neurons display oscillated PKA activity (Dunn & Feller, 2008) on the timescale of ~40 s (Dunn et al., 2006), suggesting that PKA activity may directly regulate the wave frequency. Remarkably, PKA activity is high during the inter-wave interval, implying that certain PKA substrates are involved in decreasing the SAC release to cause wave quiescence. Our previous study revealed that the core exocytotic molecule SN25b serves as a PKA substrate in SACs, further regulating wave activity during development (Hsiao et al., 2019). Particularly, PKA-mediated SN25b phosphorylation at T138 inhibits the transmission from developing SACs, thus down-regulating the wave spatial–temporal properties. In this study, we found that CSPα also serves a PKA substrate in SACs. By contrast, PKA-mediated CSPα phosphorylation at S10 maintains the transmission from developing SACs, serving an up-regulatory role in maintaining the wave frequency, without altering the wave spatial properties. Thus, through dynamic changes in the PKA-mediated phosphorylation of downstream substrates, the release from SACs and cholinergic waves can be rapidly modulated, leading to precise regulation of activity-dependent synaptic refinement for the entire visual circuits.

In this study, we found that CSPα-S10 phosphodeficiency dampens the speed (rather the amount) of input signals that RGCs receive during waves, without changing the RGC intrinsic excitability. These results suggest that the effects are due to the alteration in the rate of SAC exocytosis. Previous work in cell lines showed that the phosphorylation state of CSPα-S10 regulates the rate of exocytosis (Chiang et al., 2014) as well as the interaction of CSPα with Syt I (Evans & Morgan, 2002) or Stx (Chiang et al., 2014). Remarkably, Syt I is also found to regulate the frequency of cholinergic waves *via* SACs (Chiang et al., 2012). Moreover, CSPα is a chaperone protein, which is important for degradation of misfolded SN25 (Sharma et al., 2011, 2012). Whether or how the phosphorylation state of CSPα-S10 may regulate the SAC exocytosis requires further investigation.

During this stage, non-SAC cells, such as RGCs, may also express CSP that contributes to the CSP immunoreactivity observed in the IPL (Fig. 1A and 1D). These developing RGCs receive acetylcholine (ACh) and *γ*-amino butyric acid (GABA) released from SACs and thus participate in the propagation of cholinergic waves. At present, it remains unknown if CSP plays any role in modulating the function of RGCs. However, in various types of cells, CSPα also interacts and modulates many cellular proteins, such as G protein subunit (Gorenberg & Chandra, 2017). Therefore, CSPα in RGCs may also participate in the propagation of cholinergic waves through its interacting proteins that are involved in cellular functions, which awaits further characterization.

Cholinergic waves propagate through developing retinas and the thalamus to the visual cortex, essential for refining the visual sensory map (Blankenship & Feller, 2010; Ackman et al., 2012). Misconnected circuits are common features of neurodevelopmental disorders such as schizophrenia and autism. Therefore, the effects of PKA-mediated CSPα phosphorylation would not only provide insights into visual circuit refinement, but also shed the light into circuit development as well as the etiology of neurodevelopmental diseases (Lewis & Levitt, 2002).
